# Advanced Parameters of Myocardial Strain and Cardiac Biomarkers Indicate Subclinical Systolic Myocardial Dysfunction in Patients with Systemic Lupus Erythematous

**DOI:** 10.3390/biomedicines12112638

**Published:** 2024-11-19

**Authors:** Nikolaos P. E. Kadoglou, Alexandriani Dimopoulou, Ioannis Korakianitis, Konstantinos Parperis

**Affiliations:** Medical School, University of Cyprus, 215/6 Old Road Lefkosias-Lemesou, Aglatzia CY 2029, Nicosia, Cyprusparperis.konstantinos-marinos@ucy.ac.cy (K.P.)

**Keywords:** systemic lupus erythematous, echocardiography, myocardial work, speckle tracking echocardiography, high sensitivity troponin, natriuretic peptide

## Abstract

**Background**: Systemic lupus erythematosus (SLE) is characterized by inflammation and cardiovascular complications. Our study aimed to investigate subclinical and early indicators of systolic myocardial dysfunction in SLE patients using advanced echocardiographic methods and biomarkers. **Methods**: In this cross-sectional study, we enrolled 102 SLE patients without known cardiac impairment and 51 healthy controls. Demographics, disease characteristics, laboratory results, disease activity (SLEDAI), and organ damage (SDI) indices were recorded. Left ventricular global longitudinal strain (GLS) and myocardial work indices were assessed by utilizing speckle tracking echocardiography. In addition, high-sensitivity C-reactive protein (hsCRP), high-sensitivity troponin (hsTn), and N-terminal-pro B-type natriuretic peptide (NT-proBNP) levels were measured in blood samples. **Results**: In comparison with controls, SLE patients had significantly higher GLS (−19.94 ± 2.71% vs. −21.15 ± 1.55%, *p* < 0.001) and global wasted work (GWW) (94 ± 71 mmHg% vs. 71 ± 49 mmHg%, *p* = 0.025). Notably, NT-proBNP and hsTn were threefold and twofold higher in the SLE group compared with the control group, respectively (*p* < 0.001). Within the SLE cohort, in patients with at least moderate disease activity (SLEDAI ≥ 4), both biomarkers were significantly more elevated than those with low disease activity (SLEDAI < 4). Notably, hsTn levels remained within the normal range. **Conclusions**: Advanced echocardiographic parameters combined with specific biomarkers have a promising role in detecting systolic dysfunction in SLE patients, potentially enabling timely interventions to mitigate cardiovascular risk

## 1. Introduction

Systemic lupus erythematosus (SLE) is a prototypic autoimmune disease characterized by systemic inflammation and dysfunction of various organs, including the cardiovascular system [1]. Cardiac manifestations are commonly observed in SLE patients and can involve any part of the heart, including the myocardium, valves, conduction system, pericardium, and coronary arteries [2]. Myocardial disease can be present in 8–14% of SLE patients, and the nonspecific nature of associated signs and symptoms that are often confused with SLE clinical manifestations may lead to underdiagnosis of myocardial dysfunction, heightening the risk of morbidity and mortality [3]. Heart failure (HF) is a common complication in SLE patients, negatively impacting their prognosis [4]. While HF with reduced ejection fraction (HFrEF) occurs less frequently, the prevalence of subclinical left ventricular (LV) systolic dysfunction or HF with preserved ejection fraction (HFpEF) is speculated to be higher in the SLE population. The connection between SLE and HF is primarily attributed to immune-mediated mechanisms and the presence of traditional cardiovascular risk factors such as hypertension and hyperlipidemia [5].

The introduction of speckle tracking echocardiography (STE) has conferred a great advantage in the early detection of subtle reduction in LV systolic function [6,7]. It has been demonstrated that the impairment of LV deformation becomes detectable before the development of an overt systolic dysfunction, especially among patients with auto-immune diseases [8]. To date, a limited number of studies have investigated the presence of subtle systolic dysfunction in SLE patients utilizing STE [9,10]. The latter studies implicate the early development of myocardial dysfunction before becoming apparent clinically or with the classical echocardiographic indices, like LV ejection fraction (LVEF), in comparison with healthy controls.

The main disadvantage of global longitudinal strain (GLS) calculation is its dependence on blood pressure measurement. A novel advancement in STE is its ability to calculate myocardial work (MW)—proposed as an early marker of cardiac damage—independent of blood pressure level. MW is an evolving echocardiographic tool linked with the pathophysiology of myocardial function, but more studies are required to clarify its clinical impact and its advantages over STE and to set its limitations [11]. A recently published study has demonstrated that MW is a more sensitive tool for detecting subclinical LV systolic dysfunction in the SLE population [12]. Unambiguously, more studies are required for comparative evaluation.

Cardiac biomarkers such as natriuretic peptides and troponin have long been used as tools for diagnostic and prognostic purposes among patients with suspected or established HF, respectively [13]. Limited studies have documented elevated blood concentrations of both those biomarkers in SLE patients [14]. Therefore, a diagnostic strategy that integrates STE, MW assessment, and cardiac biomarkers could enhance the early detection of subclinical systolic myocardial dysfunction in SLE patients. This integrated strategy would enable clinicians to initiate treatment earlier, potentially leading to better patient outcomes.

This study examined the hypothesis that SLE negatively impacts LV myocardial systolic function at a subclinical level in SLE patients without overt cardiovascular disease (CVD). It had the following aims: (1) to evaluate LV myocardial systolic function using STE, MW, and cardiac biomarkers (natriuretic peptides and troponin), (2) to explore the relationship between those echocardiographic parameters and biomarkers and indices of disease activity, organ damage, or other clinical parameters.

## 2. Materials and Methods

### 2.1. Participants

We recruited 102 adult patients diagnosed with SLE from two centers over the period from September 2022 to October 2023. SLE diagnosis was based on the Systemic Lupus International Collaborating Clinics (SLICC) 2012 classification criteria [15]. The history of any SLE-related complication was retrieved from the medical records. Among the exclusion criteria were concurrent cardiovascular diseases such as coronary artery disease (CAD), HFrEF, peripheral artery disease, or other cardiomyopathies and patients with concomitant significant kidney failure (end-stage renal disease on dialysis) or moderate to severe liver dysfunction with a Child-Pugh score of B or C, or recent infection, surgery, or trauma which might have increased the inflammatory burden. We excluded patients with chronic diseases associated with poor prognosis, like those with a direct impact on life expectancy and quality of life, such as advanced heart failure, untreatable cancer, or progressive neurological diseases such as advanced Parkinson’s disease or multiple sclerosis. Additionally, patients with poor-quality echocardiographic images, which could question the interpretation of echocardiography analysis, were not included in the final enrolment.

Furthermore, 51 sex- and age-matched healthy individuals without any chronic disease were recruited as controls. We selected a 2:1 matching ratio to increase study efficiency and reduce the confounding effects of matching factors. The study adhered to the ethical guidelines of the Declaration of Helsinki. The study obtained approval from the national bioethical committee (EEBK/EP/2019/03). Before entering the study, all participants provided signed informed consent forms.

### 2.2. Study Design

We conducted an observational, cross-sectional study. A complete medical history was obtained, including demographic data, disease duration, organ involvement, current or previous treatments, and indices of disease activity or organ damage. The disease activity was analyzed using the Safety of Estrogens in Lupus Erythematosus National Assessment–SLEDAI instrument score (SELENA-SLEDAI), and organ damage was analyzed using the SLICC/ACR Damage Index (SDI) [16,17]. To further assess the impact of disease activity on the examined parameters, we initially subdivided the SLE group into subgroups with high disease activity (SELENA-SLEDAI ≥ 10), moderate disease activity (4 ≤ SELENA-SLEDAI < 10), and low disease activity (SELENA-SLEDAI < 4) based on a recent study [12]. That cut-off value was used for comparative reasons. Since only 3 patients appeared with high disease activity, we ended up with 2 subgroups: (a) with at least moderate disease activity (SELENA-SLEDAI ≥ 4) and (b) with low disease activity (SELENA-SLEDAI < 4). We also comparatively evaluated patients with organ damage and no damage using the cutoff value SDI ≥ 1 [12]. SLE-related complications, such as nephritis and pericarditis, were defined according to the SLICC criteria. It is well-known that those complications may adversely affect the clinical course of SLE patients [18].

In the context of medical history, we recorded medications and cardiovascular risk factors, including active smoking, hypertension, diabetes mellitus (DM), dyslipidemia, and family history of premature CAD. We also measured blood pressure, body weight and height, and body mass index (BMI) was calculated. Further, all eligible participants underwent rest echocardiography conducted by an experienced cardiologist, and offline image analysis for speckle tracking and MW was performed. Subsequently, blood samples were collected for the measurement of high-sensitivity C-reactive protein (hsCRP), high-sensitivity cardiac troponin I (hs-cTnI), and N-terminal-pro B-type natriuretic peptide (NT-proBNP). The echocardiographic and laboratory methods are described in the following sections.

### 2.3. Global Longitudinal Strain (GLS) and Myocardial Work (MW)

We examined the LV myocardial deformation by calculating the LV GLS formula using the following steps: During breath-holding, we recorded 2 consecutive cardiac cycles of the 4-chamber, 2-chamber, and 3-chamber apical views. The frame rate frequency was above 60 frames/s. Longitudinal strain was measured from 3 apical views, with each wall subsequently divided into 3 segments (basal, mid, and apical), resulting in a total of 17 segmental strain curves. This analysis was performed using the EchoPAC Version 203 software package (GE Vingmed Ultrasound, Oslo, Norway). GLS was calculated as the average value of the three apical strain peak values. Two experienced cardiologists made the calculations, blinded to patients’ medical history. The intra and inter-observer reliability of strain analysis by our group has been previously reported and found to be very low (<2.5%) [19].

Blood pressure was considered equivalent to LV pressure. To build up a global LV pressure–strain loop adjusted on valvular timing events, the vendor-specific software integrated a global LV strain curve with a non-invasively predicted LV pressure curve. The MW was quantified by computing the region myocardial shortening rate and multiplied by LV pressure during the LV isovolumic contraction and ejection period. The regional constructive work (CW) was generated during segmental shortening, while the regional segmental elongation comprised the regional wasted work (WW). We also calculated: (1) global work index (GWI, mmHg%): total MW within the area enclosed in the LV pressure-strain loop (from mitral valve closure through to mitral valve opening); (2) global constructive work (GCW, mmHg%): total MW of 17 segments generated during myocardial shortening in systole and lengthening in isovolumic relaxation; (3) global wasting work (GWW, mmHg%): total MW of 17 segments generated during myocardial lengthening in systole and shortening in isovolumic relaxation; and (4) global wasting efficiency (GWE, %): The ratio of GCW/(GCW + GWW) [20,21].

### 2.4. Blood Assays

Blood samples were promptly collected after overnight fasting and were subjected to centrifugation, after which the resulting serum was stored in a deep freezer at −80 °C. The measurements of serum hsCRP, hs-cTnI, and NT-proBNP and were conducted using the Alinity analyzer from Abbott Diagnostics (Abbott Park, Libertyville Township, IL, USA). This process involved a two-step immunoassay conducted in human serum, utilizing chemiluminescent microparticle immunoassay (CMIA) technology. According to the manufacturer’s specifications, the precision of the hs-cTnI and NT-proBNP assay at low concentrations is adequate, enabling the assessment of various thresholds with a coefficient of variation (CV) of 3.2% in our laboratory.

The creatinine blood levels were measured using an automatic enzymatic analyzer (Olympus AU560, Hamburg, Germany).

### 2.5. Statistical Analysis

Before conducting the analysis, the normal distribution of continuous variables was assessed using the Kolmogorov–Smirnov test. Because a normal distribution was found, non-parametric tests were not necessary. Continuous variables were presented as mean ± SD, and group comparisons were carried out using Student’s *t*-test. Categorical variables were expressed as numbers or frequencies (%) and were compared using the Chi-square test. The assessment of SLE activity and organ damage involved stratification into subgroups, as previously described in the “study design” section.

To investigate the relationship of parameters of LV systolic function (GLS, MW indices) and biomarkers (hs-cTnI and NT-proBNP) with clinical parameters and SLE activity, univariate analysis was performed using Pearson correlation. Subsequently, a backward multiple regression analysis was conducted. The data analysis was carried out using SPSS version 25.0, with statistical significance set at a *p*-value of ≤0.05.

## 3. Results

A total of 126 patients with SLE were selected for the study. Following a comprehensive clinical and echocardiographic assessment, 102 consecutive patients diagnosed with SLE (mean age: 51, 90% women) were considered eligible for participation in this study. A significant proportion of SLE patients were on prednisolone (41.5%) and hydroxychloroquine (82%). Based on medical records, 24.5% and/or 13.7% of SLE patients reported nephritis and/or pericarditis, respectively, in the past. None of the SLE patients had a history of cardiovascular disease (e.g., myocardial infarction, myocarditis), significant kidney impairment, or acute myocarditis. Almost 25% of SLE patients were on medications for hypertension, and a similar percentage had dyslipidemia (Table 1).

Additionally, a control group consisting of 51 individuals (mean age: 50 years, 88% women) was included in the study. Those healthy control participants did not have any chronic illness and were not on any chronic medication regimen. The comparative evaluation of those groups showed no significant differences between them concerning gender distribution, vital signs, or BMI. Regarding the assessment of SLE activity, all relevant parameters were evaluated. Using the Systemic Lupus Erythematosus Disease Activity Index (SELENA-SLEDAI), 39 patients were identified as having moderate or high disease activity (SELENA-SLEDAI ≥ 4), and when employing the SDI, 19 patients were categorized as having organ damage (SDI ≥ 1).

### 3.1. Echocardiographic Findings and Cardiac Biomarkers

Most of the classical echocardiographic indices did not significantly differ between groups, as shown in Table 1. However, a small proportion of SLE patients (4%) exhibited diastolic dysfunction (defined as E/A < 0.8, E/E’ > 8, and left atrium dilatation), and 4% had left ventricular hypertrophy (LVH). Surprisingly, SLE patients had lower levels of tricuspid annular plane systolic excursion (TAPSE) (*p* < 0.001), but those levels remained within the normal range compared to controls. Concerning the novel echocardiographic markers, SLE patients exhibited higher values of GLS (*p* < 0.001) and GWW (*p* = 0.025) compared with controls. No significant differences were observed in the rest of the MW parameters.

hs-cTnI and NT-proBNP blood concentrations were twofold and almost threefold higher, respectively, in SLE patients compared to controls (*p* < 0.001). Despite the hs-cTnI elevation, its level remained within the normal range in all SLE patients. In the SLE group, patients with previous nephritis and/or pericarditis tended to have higher hs-cTnI levels (3.53 pg/mL vs. 2.78 pg/mL, *p* = 0.126) and NT-proBNP levels (170 ± 100 pg/mL vs. 148 ± 100 pg/mL, *p* = 0.293) than complication-free counterparts, but those differences did not reach statistical significance.

### 3.2. Comparison Based on Disease Activity or Organ Damage

When applying the cutoff value (SELENA-SLEDAI ≥ 4), we observed non-significant differences in LVEF and novel echocardiographic findings between subgroups, apart from GCW, which was reduced in the subgroup with higher disease activity. Furthermore, patients with at least moderate disease activity had significantly higher hs-cTnI and NT-proBNP levels. Non-significant differences were observed in the rest of the parameters between disease activity-based subgroups. All clinical, echocardiographic, and biochemical characteristics of both subgroups are depicted in Table 2.

The subdivision of the SLE cohort based on SDI levels did not reveal significant differences between subgroups, perhaps due to the unequivocal distribution of participants (Table 3).

### 3.3. Correlations

In the univariate analysis of cardiac biomarkers, we observed notable correlations. Hs-cTnI displayed significant associations with several key variables, including the SELENA-SLEDAI, SDI, GLS, and NT-proBNP (*p* < 0.05). Similarly, NT-proBNP exhibited significant correlations with SLEDAI, SDI, GLS, Left Atrial Volume Index (LAVI), and troponin (*p* < 0.05). Variables with significant univariate associations with either hs-cTnI or NT-proBNP entered the multivariate regression analysis. SLEDAI, GLS, and NT-proBNP remained independent predictors of hs-cTnI as the dependent variable (R^2^ = 0.242, *p* = 0.011). In parallel, SELENA-SLEDAI, GLS and troponin independently predicted NT-proBNP levels among SLE patients (R^2^ = 0.371, *p* = 0.021). The results are presented in Table 4 and Table 5.

We also examined the relationships between disease activity (expressed by SELENA-SLEDAI) and organ damage (expressed by SDI) and the rest of the variables. It is noteworthy that, despite an initial univariate correlation between SELENA-SLEDAI and GCW (r = −0.224, *p* = 0.034), this association did not persist when subjected to the multiple linear regression analysis.

## 4. Discussion

In the present study, the comparative evaluation of SLE patients without CVD versus healthy controls revealed impaired GLS and GWW index of MW in SLE patients, indicating subtle cardiac dysfunction. Elevated circulating levels of both hs-cTnI and NT-proBNP were detected in SLE patients, even though the elevated hs-cTnI concentrations remained within the normal range. Those differences in cardiac biomarkers may underscore the potential cardiac involvement in SLE despite the absence of overt CVD. Furthermore, both cardiac biomarkers, hs-cTnI and NT-proBNP, demonstrated independent associations with GLS in SLE patients. In the subgroup of SLE patients with at least moderate disease activity (SELENA-SLEDAI ≥ 4), we observed a significant reduction in GCW and a considerable elevation of both hs-cTnI and NT-proBNP levels compared to the low disease activity counterparts.

One of the major complications of SLE associated with poor prognosis is cardiac systolic dysfunction [9], which is primarily associated with the duration and severity of the disease [22]. There are two main underlying causes related to systolic dysfunction. First, the reasons for premature CAD include precipitated atherosclerosis, thrombosis, endothelial damage, inflammation, renal impairment, hypertension, dyslipidemia, and corticosteroid administration. A second cause is myocarditis, induced by inflammatory insults such as immune-mediated mechanisms. Previous studies have reported a very low incidence of HFrEF among SLE patients [23], while diastolic dysfunction may be more common [24]. In our study, we excluded patients with impaired systolic function, like HFrEF. It is worth noting that the sensitivity of classical echocardiographic parameters in detecting minor systolic dysfunction is very low, especially in the early stages of CAD or in patients with myocarditis spread across a limited area. Regarding the impact of SLE on myocardial function, there is a growing body of evidence indicating that subclinical LV dysfunction among SLE patients is a common finding [12,13]. This dysfunction is typically identified through GLS measurements despite preserved LVEF [9,25]. In line with a recent meta-analysis of nine studies [26], we confirmed lower GLS values in SLE patients compared with age- and sex-matched healthy controls. Notably, our SLE cohort had a low cardiovascular risk profile, with no previous atherothrombotic cardiovascular disease or myocarditis recorded. In addition, a small proportion of patients had nephritis or pericarditis in the past without any remaining disorders. What adds significant value to our results is the detection of reduced GLS in otherwise cardiac-uncomplicated SLE patients. This finding indicates the early involvement of SLE in myocardial dysfunction, which may alter the management of those patients. Lower strain values indicate subclinical impairment of the myocardium and have been linked to a higher incidence of cardiovascular adverse events, as observed in both HFpEF [27] and SLE populations [28]. However, the link between STE and clinical endpoints in the SLE cohort needs further investigation.

The dependence of GLS on afterload can affect its accuracy in assessing LV systolic function. However, the calculation of the strain–pressure loop using the recently proposed MW indices may overcome this disadvantage of GLS. Growing research has shed light on the clinical applicability of MW across a wide spectrum of cardiomyopathies [29,30]. In a previous study on SLE patients conducted by He W et al., GWW and GWE appeared with abnormal values, along with an independent association of GWE with SLE activity measured using the SLEDAI-2K index [12]. To our knowledge, this is the second study reporting higher GWW in SLE patients than in controls. The markedly elevated GWW in SLE may represent a compensatory mechanism to maintain LV systolic function in the face of increased afterload. This increase in GWW points towards the presence of systolic dysfunction at an early stage. While approximately one-fifth of our SLE patients had hypertension, in most of them, blood pressure was well-controlled, and only 4% presented with LV hypertrophy. Consequently, we did not anticipate a substantial impact of hypertension without LVH on the MW indices.

We further examined whether disease activity may drive the considerable difference in GLS and GWW within the SLE group. We realized that the difference was not attributed to the current disease activity. In this context, we failed to demonstrate an independent relationship between SELENA-SLEDAI and GLS or any MW index in the multiple regression analysis, in contrast to previous results from He et al. [12]. Within the SLE group, patients with more active disease had lower GCW, while the rest of the classical and novel echocardiographic indices were not affected by the level of disease activity. Regarding the limitations of the GCW calculation, our results do not provide robust evidence about the interplay between disease activity and myocardial strain. In contrast, previous authors reported a linear relationship between myocardial strain impairment and disease activity [31,32]. Plausible explanations for this inconsistency derive from the inherent limitations of the SELENA-SLEDAI score in quantifying disease activity. Compared with previous studies, our cohort had a more favorable cardiovascular risk profile since none had known cardiovascular disease or significant chronic kidney dysfunction. A small percentage of patients had prior nephritis/pericarditis, and our patients had a smaller duration of SLE. In addition to this, there was a lower average degree of disease activity, which was predominantly moderate, since only 3% appeared with high disease activity (SELENA-SLEDAI ≥ 10). We believe that a long follow-up and the recruitment of patients with cardiovascular complications and more severe disease activity could clarify whether the inflammatory burden (disease activity) could directly affect myocardial function even at a subclinical level. The presence of systemic inflammation, as is characteristic of SLE, may play a more critical role in early myocardial dysfunction. Recent insights have linked autoantibodies such as lupus anticoagulants, anti-cardiolipin, and anti-ds DNA with myocardial damage, either directly through binding to myocardial cells or indirectly through immune complex deposition [33,34]. This is supported by findings that demonstrate a correlation between immune complexes and cardiac manifestations in SLE patients. Chronic systemic inflammation can lead to the release of pro-inflammatory cytokines (e.g., TNF-α), which have been shown to contribute to myocardial stress and fibrosis [35]. Elevated levels of type I interferon have been implicated in promoting inflammatory processes in SLE, which can exacerbate cardiovascular complications [36]. The IFN-I pathway has been specifically noted for its role in upregulating pro-inflammatory responses, potentially leading to impaired endothelial function, accelerated atherosclerosis, and cardiac dysfunction. Finally, the index of organ damage, the SDI, did not differentiate the echocardiographic findings at all.

In comparison with previous studies [37], one could argue that our SLE patients did not exhibit systolic dysfunction because of the preserved LVEF and the slight reduction in GLS levels, which remained within the normal range (<−18%) [28]. This raises the question about the clinical significance of the differences in myocardial strain between SLE and healthy control groups. To limit the potential random effects of participant selection, we simultaneously examined the circulating levels of biomarkers of myocardial injury (hs-cTnI) and myocardial overload (NT-proBNP). To the best of our knowledge, this is the third study examining the concentrations of troponin in SLE patients. In a smaller study of 63 SLE patients with a low cardiovascular risk profile like ours, high hs-troponin levels were associated with the presence of carotid atherosclerotic lesions [38]. In another study, Sabio JM et al. (2023) demonstrated higher arterial stiffness in SLE patients with detectable values of hs-troponin compared with controls [39]. Notably, both studies used the dichotomous values of detectable and non-detectable hs-troponin levels for analysis. In our study, we analyzed hs-cTnI as a continuous variable, thereby increasing its sensitivity. Although the observed values of hs-cTnI in our study remained within the normal range, they were two times higher than those in the control group, indicating a potential myocardial involvement.

Recently, natriuretic peptides have emerged as biomarkers of cardiac disorders in SLE patients [40]. From a clinical perspective, the elevation of natriuretic peptides has traditionally been interpreted as a result of SLE-induced cardiac complications, such as pulmonary hypertension or HFrEF [41]. In the context of the low cardiovascular risk profile of our SLE cohort, the observed remarkable elevation in NT-proBNP is of paramount importance since it is by far the most sensitive biomarker of increased intra-cardiac pressure and overt cardiac dysfunction [42]. Even after excluding patients with prior lupus nephritis, a considerable difference in NT-proBNP levels remained between SLE patients and healthy controls. Like hs-cTnI, NT-proBNP is a well-established independent predictor of future cardiovascular events [43]. Therefore, utilizing a combination of these biomarkers in routine clinical assessments has the potential to better evaluate cardiovascular risk in SLE patients, even in the early stages of their disease.

Both hs-cTnI and NT-proBNP levels were influenced by disease activity, aligning with prior studies reporting higher levels of those biomarkers in patients with autoimmune diseases without cardiac symptoms [44,45]. Although hs-cTnI provides significantly higher sensitivity, it comes at the cost of decreased specificity. The upper reference limits (URLs) can introduce variability in studies investigating myocardial injury rather than ischemia [46]. The fact that all hs-cTnI values fell within the normal range makes us suspicious of the clinical significance of its link with subtle cardiac dysfunction. After excluding confounding comorbidities, measurements of hs-cTnI below the 99th percentile may still maintain their diagnostic [47] and prognostic value [48]. After the exclusion of acute or subacute myocarditis or pericarditis, we found that the increased levels of hs-cTnI and NT-proBNP in our SLE patients were linked to GLS and disease activity. This suggests that cardiac biomarkers can help to detect and quantify ongoing myocardial injury and subtle cardiac dysfunction in SLE patients due to disease activity. Determining specific cutoff values for these biomarkers will assist in detecting SLE-related cardiac effects, but large prospective trials are unambiguously required.

There are some limitations in the present study. First, the small sample size and the cross-sectional design may introduce some bias into our results. We anticipate that future prospective studies will confirm the prognostic value of our findings. Second, we did not have access to a more sensitive imaging modality, like cardiac magnetic resonance (CMR), which could provide a more detailed assessment of the myocardial texture and function. If CMR was available to all participants, we could objectively detect cases of past myocarditis. Furthermore, the present study was a two-center study, and larger studies are needed to provide insights into the clinical implementation of our findings. Finally, we did not have access to comprehensive medication records for all participants, which prevented us from including these data in our analysis.

## 5. Conclusions

In conclusion, our study revealed that SLE patients without known cardiovascular disease had subtle systolic impairment compared with healthy controls, even though the classical echocardiographic parameters of systolic and diastolic function did not differ from those in healthy controls. One of the most striking findings of our study was the remarkable elevation of circulating levels of both hs-cTnI and NT-proBNP, which remained unaffected by co-morbidities and age, paralleling higher GLS and GWW levels in SLE patients. Notably, those biomarkers were independently associated with disease activity, suggesting that they could serve as valuable indicators of myocardial insult in cases of moderate or high disease activity. Future studies will investigate the prognostic implications of our findings and the potential integration into the management of SLE patients.

## Figures and Tables

**Table 1:** Comparison between patients with SLE and healthy controls.

	SLE Patients (n = 102)	Healthy Controls (n = 51)	*p*-Value
Age (years)	51 ± 15	50 ± 7	0.352
Males/females (n)	10/92	6/45	0.720
Hypertension (n)	25 (25.5%)	0	-
Dyslipidemia (n)	25 (25.5%)	0	-
Diabetes (n)	3 (2.9%)	0	-
Nephritis (n)	25 (25.5%)	0	-
Pericarditis (n)	14 (13.7%)	0	-
Duration SLE (years)	13 ± 8	-	-
SLEDAI ≥ 4 (n)	39 (38.2%)	-	-
SDI ≥ 1 (n)	19 (18.6%)	-	-
BMI (kg/m^2^)	25.45 ± 4.11	26.12 ± 4.54	0.218
SBP (mmHg)	135 ± 17	131 ± 13	0.189
DBP (mmHg)	81 ± 13	82 ± 8	0.833
HR (bpm)	73 ± 11	72 ± 9	0.703
Echocardiography			
LVEF (%)	65 ± 7	66 ± 7	0.205
E/A ratio	1.22 ± 0.53	1.19 ± 0.29	0.753
E/E’ ratio	7.37 ± 5.32	6.11 ± 1.56	0.134
TAPSE (cm)	2.2 ± 0.3	2.5 ± 0.4	<0.001
RVS’ (m/s)	0.77 ± 2.48	0.14 ± 0.02	0.133
TRVmax (m/s)	2.17 ± 0.41	2.71 ± 2.88	0.159
LAVI (mL/m^2^)	33.1 ± 17.3	34.4 ± 13.5	0.669
GLS (%)	−19.84 ± 2.51	−21.35 ± 1.25	<0.001
GWI (mmHg%)	2072 ± 421	2080 ± 346	0.899
GWW (mmHg%)	94 ± 71	71 ± 49	0.025
GCW (mmHg%)	2401 ± 475	2397 ± 365	0.960
GWE ratio (%)	95.64 ± 2.73	96.33 ± 2.26	0.143
Biomarkers			
hs-cTnI (pg/mL)	3.33 ± 2.10	1.56 ± 1.02	<0.001
NT-proBNP (pg/mL)	163.71 ± 86.82	58.55 ± 23.87	<0.001
Creatinine (mg/dL)	1.2 ± 0.3	0.7 ± 0.2	0.171

Continuous variables are presented as mean ± standard deviation (SD). Categorical variables are presented as number of participants (% of the group). Abbreviations: DBP, Diastolic blood pressure; E/A ratio, E transmitral flow velocity/A transmitral flow velocity; E/E’, E transmitral flow velocity/E’ tissue; GLS, Global longitudinal strain; GCW, Global constructive work; GWE, Global work efficiency; GWI, Global work index; GWW, Global wasted work; HR, Heart rate; hs-cTnI, high-sensitive cardiac troponin I; LAVI, Left atrial volume index; LVEF, Left ventricular ejection fraction; n, Number of participants; NT-proBNP, N-terminal pro–B-type natriuretic peptide; RVS’, Right ventricular systolic excursion velocity by tissue Doppler; SBP, Systolic blood pressure; SDI, SLICC/ACR Damage Index; SLE, Systemic lupus erythematosus; SLEDAI, Systemic Lupus Erythematosus Disease Activity Index; TAPSE, Tricuspid annular plane systolic excursion; TRVmax, Maximal tricuspid regurgitation velocity.

**Table 2:** Comparison within SLE group of patients with at least moderate disease activity (SLEDAI ≥ 4) vs. low disease activity (SLEDAI < 4).

	Subgroup A (SLEDAI < 4) n = 63	Subgroup B (SLEDAI ≥ 4) n = 39	*p*-Value
Age (years)	51 ± 17	51 ± 12	0.827
Duration (years)	16 ± 14	13 ± 11	0.439
Hypertension (n)	13 (20.6%)	12 (30.4%)	0.091
Dyslipidemia (n)	14 (22.2%)	11 (28.2%)	0.287
Diabetes (n)	2	1	-
Nephritis (n)	14 (22.2%)	11 (28.2%)	0.287
Pericarditis (n)	7 (11.1%)	7 (17.9%)	0.074
BMI (Kg/m^2^)	24.97 ± 4.52	25.96 ± 4.68	0.191
SBP (mmHg)	135 ± 14	137 ± 16	0.212
DBP (mmHg)	81 ± 12	82 ± 14	0.959
Echocardiography			
LVEF (%)	66 ± 6	65 ± 6	0.743
GLS (%)	−20.11 ± 2.99	−19.52 ± 2.30	0.327
GWI (mmHg%)	2065 ± 453	2086 ± 365	0.806
GWW (mmHg%)	93 ± 69	95 ± 45	0.858
GCW (mmHg%)	2489 ± 398	2231 ± 356	0.049
GWE ratio (%)	91.30 ± 4.35	97.91 ± 2.19	0.303
Biomarkers			
hs-cTnI (pg/mL)	2.89 ± 1.50	4.01 ± 2.76	0.008
NT-proBNP (pg/mL)	122.2 ± 70.4	200.3 ± 112.6	<0.001
Creatinine (mg/dL)	1.1 ± 0.3	1.3 ± 0.4	0.789

Continuous variables are presented as mean ± standard deviation (SD). Categorical variables are presented as number of participants (% of the group). Abbreviations: BNP, Brain natriuretic peptide; BMI, Body-mass index; DBP, Diastolic blood pressure; GLS, Global longitudinal strain; GCW, Global constructive work; GWE, Global work efficiency; GWI, Global work index; GWW, Global wasted work; hs-cTnI, high-sensitive cardiac troponin I; LVEF, Left ventricular ejection fraction; n, Number; NT-proBNP, N-terminal pro–B-type natriuretic peptide; SBP, Systolic blood pressure; SLEDAI, Systemic Lupus Erythematosus Disease Activity Index.

**Table 3:** Comparison within SLE group of patients with organ damage (SDI ≥ 1) or not (SDI < 1).

	Subgroup A (SDI < 1) n = 83	Subgroup B (SDI ≥ 1) n = 19	*p*-Value
Age (years)	51 ± 12	52 ± 14	0.708
Duration (years)	12 ± 9	15 ± 10	0.551
Hypertension (n)	18 (21.7%)	7 (36.8%)	0.066
Dyslipidemia (n)	20 (24.1%)	5 (26.3%)	0.890
Diabetes (n)	3	0	-
Nephritis (n)	19 (22.9%)	6 (31.6%)	0.091
Pericarditis (n)	10 (13.3%)	4 (21%)	0.054
BMI (Kg/m^2^)	24.67 ± 4.57	25.73 ± 4.84	0.088
SBP (mmHg)	135 ± 18	138 ± 12	0.158
DBP (mmHg)	81 ± 14	81 ± 12	0.967
LVEF (%)	67 ± 7	64 ± 10	0.432
Echocardiography			
GLS (%)	−19.92 ± 2.31	−19.38 ± 2.40	0.197
GWI (mmHg%)	2075 ± 253	2099 ± 541	0.912
GWW (mmHg%)	92 ± 73	95 ± 67	0.864
GCW (mmHg%)	2445 ± 501	2301 ± 489	0.112
GWE ratio (%)	93.16 ± 5.35	97.19 ± 5.22	0.603
Biomarkers			
Troponin (pg/mL)	2.82 ± 1.90	4.52 ± 2.52	0.076
NT-proBNP (pg/mL)	140.2 ± 85.3	204.3 ± 91	0.105
Creatinine (mg/dL)	1.1 ± 0.2	1.3 ± 0.3	0.789

Continuous variables are presented as mean ± standard deviation (SD). Categorical variables are presented as number of participants (% of the group). Abbreviations: BNP, Brain natriuretic peptide; BMI, Body-mass index; DBP, Diastolic blood pressure; GLS, Global longitudinal strain; GCW, Global constructive work; GWE, Global work efficiency; GWI, Global work index; GWW, Global wasted work; LVEF, Left ventricular ejection fraction; n, Number; SBP, Systolic blood pressure; SLEDAI, Systemic Lupus Erythematosus Disease Activity Index.

**Table 4:** Associations between high-sensitive cardiac troponin I (dependent variable) and other variables in SLE patients.

Characteristics	Univariate Analysis		Multivariate Analysis	
	β (SE)	*p*	β (SE)	*p*
NT-proBNP	0.511 (0.232)	0.002	0.237 (0.095)	0.029
SELENA-SLEDAI	0.312 (0.121)	<0.001	0.210 (0.068)	0.012
GLS	−0.488 (0.113)	<0.001	−0.322 (0.096)	0.004
SDI	0.110 (0.109)	0.041	0.031 (0.005)	0.789

β, beta value; SE, standard error. Abbreviations: GLS, Global longitudinal strain; NT-proBNP, N-terminal pro–B-type natriuretic peptide; SDI, SLICC/ACR Damage Index; SLEDAI, Systemic Lupus Erythematosus Disease Activity Index.

**Table 5:** Associations between NT-proBNP and other variables in SLE patients.

Characteristics	Univariate Analysis		Multivariate Analysis	
	β (SE)	*p*	β (SE)	*p*
hs-cTnI	0.589 (0.110)	0.002	0.348 (0.062)	0.002
SELENA-SLEDAI	0.282 (0.101)	0.002	0.164 (0.056)	0.031
GLS	−0.432 (0.174)	<0.001	−0.266 (0.091)	0.007
SDI	0.252 (0.156)	0.035	0.131 (0.082)	0.587
Nephritis	0.259 (0.179)	0.037	0.201 (0.138)	0.104

Abbreviations: GLS, Global longitudinal strain; hs-cTnI, high-sensitive cardiac troponin I; LAVI, Left Atrial Volume Index; SDI, SLICC/ACR Damage Index; SLEDAI, Systemic Lupus Erythematosus Disease Activity Index.

## Data Availability

The original contributions presented in the study are included in the article, further inquiries can be directed to the corresponding author.

## References

[1] Tsokos G.C. (2011). Systemic Lupus Erythematosus. N. Engl. J. Med..

[2] Miner J.J., Kim A.H. (2014). Cardiac Manifestations of Systemic Lupus Erythematosus. Rheum. Dis. Clin. N. Am..

[3] Buss S.J., Wolf D., Korosoglou G., Max R., Weiss C.S., Fischer C., Schellberg D., Zugck C., Kuecherer H.F., Lorenz H.M. (2010). Myocardial Left Ventricular Dysfunction in Patients with Systemic Lupus Erythematosus: New Insights from Tissue Doppler and Strain Imaging. J. Rheumatol..

[4] Lo Gullo A., Rodríguez-Carrio J., Gallizzi R., Imbalzano E., Squadrito G., Mandraffino G. (2020). Speckle Tracking Echocardiography as a New Diagnostic Tool for an Assessment of Cardiovascular Disease in Rheumatic Patients. Prog. Cardiovasc. Dis..

[5] Mavrogeni S., Bratis K., Markussis V., Spargias C., Papadopoulou E., Papamentzelopoulos S., Constadoulakis P., Matsoukas E., Kyrou L., Kolovou G. (2013). The Diagnostic Role of Cardiac Magnetic Resonance Imaging in Detecting Myocardial Inflammation in Systemic Lupus Erythematosus. Differentiation from Viral Myocarditis. Lupus.

[6] Kadoglou N.P.E., Papadopoulos C.H., Papadopoulos K.G., Karagiannis S., Karabinos I., Loizos S., Theodosis-Georgilas A., Aggeli K., Keramida K., Klettas D. (2022). Updated Knowledge and Practical Implementations of Stress Echocardiography in Ischemic and Non-Ischemic Cardiac Diseases: An Expert Consensus of the Working Group of Echocardiography of the Hellenic Society of Cardiology. Hell. J. Cardiol..

[7] Ikonomidis I., Pavlidis G., Kadoglou N., Makavos G., Katogiannis K., Kountouri A., Thymis J., Kostelli G., Kapniari I., Theodoropoulos K. (2022). Apremilast Improves Endothelial Glycocalyx Integrity, Vascular and Left Ventricular Myocardial Function in Psoriasis. Pharmaceuticals.

[8] Sahin S.T., Yilmaz N., Cengiz B., Yurdakul S., Cagatay Y., Kaya E., Aytekin S., Yavuz S. (2019). Subclinical Biventricular Systolic Dysfunction in Patients with Systemic Sclerosis. Eur. J. Rheumatol..

[9] Azpiri-Lopez J.R., Galarza-Delgado D.A., Garza-Cisneros A.N., Guajardo-Jauregui N., Balderas-Palacios M.A., Garcia-Heredia A., la Garza J.A.C.-D., Rodriguez-Romero A.B., la Garza R.A.R.-D., Azpiri-Diaz H. (2022). Subclinical Systolic Dysfunction by Speckle Tracking Echocardiography in Patients with Systemic Lupus Erythematosus. Lupus.

[10] Farag S.I., Bastawisy R.B., Hamouda M.A., Hassib W.A., Wahdan H.A. (2020). Value of Speckle Tracking Echocardiography for Early Detection of Left Ventricular Dysfunction in Patients with Systemic Lupus Erythematosus. J. Cardiovasc. Echogr..

[11] Dell’Angela L., Nicolosi G.L. (2024). From Ejection Fraction, to Myocardial Strain; Myocardial Work in Echocardiography: Clinical Impact and Controversies. Echocardiography.

[12] He W., Li J., Zhang P., Wan M., Xie P., Liang L., Liu D. (2023). Non-Invasive Left Ventricular Myocardial Work Identifies Subclinical Myocardial Involvement in Patients with Systemic Lupus Erythematosus. Int. J. Cardiol..

[13] Vazquez-Montes M.D.L.A., Debray T.P.A., Taylor K.S., Speich B., Jones N., Collins G.S., Hobbs F., Magriplis E., Maruri-Aguilar H., Moons K.G.M. (2020). Umbrella Protocol: Systematic Reviews of Multivariable Biomarker Prognostic Models Developed to Predict Clinical Outcomes in Patients with Heart Failure. Diagn. Progn. Res..

[14] Blachut D., Przywara-Chowaniec B., Mazurkiewicz M., Tomasik A. (2024). Assessment of Arterial Stiffness and Biochemical Markers in Systemic Lupus Erythematosus in the Diagnosis of Subclinical Atherosclerosis. J. Pers. Med..

[15] Petri M., Orbai A.M., Alarcon G.S., Gordon C., Merrill J.T., Fortin P.R., Bruce I.N., Isenberg D., Wallace D.J., Nived O. (2012). Derivation and Validation of the Systemic Lupus International Collaborating Clinics Classification Criteria for Systemic Lupus Erythematosus. Arthritis Rheum..

[16] Buyon J.P., Petri M.A., Kim M.Y., Kalunian K.C., Grossman J., Hahn B.H., Merrill J.T., Sammaritano L., Lockshin M., Alarcon G.S. (2005). The Effect of Combined Estrogen and Progesterone Hormone Replacement Therapy on Disease Activity in Systemic Lupus Erythematosus: A Randomized Trial. Ann. Intern. Med..

[17] Gladman D., Ginzler E., Goldsmith C., Fortin P., Liang M., Urowitz M., Bacon P., Bombardieri S., Hanly J., Hay E. (1996). The Development and Initial Validation of the Systemic Lupus International Collaborating Clinics/American College of Rheumatology Damage Index for Systemic Lupus Erythematosus. Arthritis Rheum..

[18] Fanouriakis A., Kostopoulou M., Alunno A., Aringer M., Bajema I., Boletis J.N., Cervera R., Doria A., Gordon C., Govoni M. (2019). 2019 Update of the Eular Recommendations for the Management of Systemic Lupus Erythematosus. Ann. Rheum. Dis..

[19] Kadoglou N.P.E., Bouwmeester S., de Lepper A.G.W., de Kleijn M.C., Herold I.H.F., Bouwman A.R.A., Korakianitis I., Simmers T., Bracke F., Houthuizen P. (2024). The Prognostic Role of Global Longitudinal Strain and Nt-Probnp in Heart Failure Patients Receiving Cardiac Resynchronization Therapy. J. Pers. Med..

[20] Russell K., Eriksen M., Aaberge L., Wilhelmsen N., Skulstad H., Remme E.W., Haugaa K.H., Opdahl A., Fjeld J.G., Gjesdal O. (2012). A Novel Clinical Method for Quantification of Regional Left Ventricular Pressure-Strain Loop Area: A Non-Invasive Index of Myocardial Work. Eur. Heart J..

[21] Boe E., Skulstad H., Smiseth O.A. (2019). Myocardial Work by Echocardiography: A Novel Method Ready for Clinical Testing. Eur. Heart J. Cardiovasc. Imaging.

[22] Yip G.W., Shang Q., Tam L.S., Zhang Q., Li E.K., Fung J.W., Yu C.M. (2009). Disease Chronicity and Activity Predict Subclinical Left Ventricular Systolic Dysfunction in Patients with Systemic Lupus Erythematosus. Heart.

[23] de Godoy M.F., de Oliveira C.M., Fabri V.A., de Abreu L.C., Valenti V.E., Pires A.C., Raimundo R.D., Figueiredo J.L., Bertazzi G.R. (2013). Long-Term Cardiac Changes in Patients with Systemic Lupus Erythematosus. BMC Res. Notes.

[24] Wislowska M., Deren D., Kochmanski M., Sypula S., Rozbicka J. (2009). Systolic and Diastolic Heart Function in Sle Patients. Rheumatol. Int..

[25] Nikdoust F., Bolouri E., Tabatabaei S.A., Goudarzvand M., Faezi S.T. (2018). Early Diagnosis of Cardiac Involvement in Systemic Lupus Erythematosus Via Global Longitudinal Strain (Gls) by Speckle Tracking Echocardiography. J. Cardiovasc. Thorac. Res..

[26] Di Minno M.N.D., Forte F., Tufano A., Buonauro A., Rossi F.W., De Paulis A., Galderisi M. (2020). Speckle Tracking Echocardiography in Patients with Systemic Lupus Erythematosus: A Meta-Analysis. Eur. J. Intern. Med..

[27] Minamisawa M., Inciardi R.M., Claggett B., Cikes M., Liu L., Prasad N., Biering-Sorensen T., Lam C.S.P., Shah S.J., Zile M.R. (2024). Clinical Implications of Subclinical Left Ventricular Dysfunction in Heart Failure with Preserved Ejection Fraction: The Paragon-Hf Study. Eur. J. Heart Fail..

[28] Zhang H., Yang C., Gao F., Hu S., Ma H. (2022). Evaluation of Left Ventricular Systolic Function in Patients with Systemic Lupus Erythematosus Using Ultrasonic Layer-Specific Strain Technology and Its Association with Cardiovascular Events: A Long-Term Follow-up Study. Cardiovasc. Ultrasound.

[29] Su Y., Peng Q., Yin L., Li C. (2022). Evaluation of Exercise Tolerance in Non-Obstructive Hypertrophic Cardiomyopathy with Myocardial Work and Peak Strain Dispersion by Speckle-Tracking Echocardiography. Front. Cardiovasc. Med..

[30] Calvillo-Arguelles O., Thampinathan B., Somerset E., Shalmon T., Amir E., Fan C.P.S., Moon S., Abdel-Qadir H., Thevakumaran Y., Day J. (2022). Diagnostic and Prognostic Value of Myocardial Work Indices for Identification of Cancer Therapy-Related Cardiotoxicity. JACC Cardiovasc. Imaging.

[31] Feng J., Zhai Z., Wang Z., Huang L., Dong S., Liu K., Shi W., Lu G., Qin W. (2021). Speckle Tracking Imaging Combined with Myocardial Comprehensive Index to Evaluate Left Ventricular Function Changes in Patients with Systemic Lupus Erythematosus. Echocardiography.

[32] Wan M., Liu D., Zhang P., Xie P., Liang L., He W. (2022). Postsystolic Shortening and Early Systolic Lengthening for Early Detection of Myocardial Involvement in Patients with Systemic Lupus Erythematosus. Echocardiography.

[33] Gustafsson J., Gunnarsson I., Borjesson O., Pettersson S., Moller S., Fei G.Z., Elvin K., Simard J.F., Hansson L.O., Lundberg I.E. (2009). Predictors of the First Cardiovascular Event in Patients with Systemic Lupus Erythematosus—A Prospective Cohort Study. Arthritis Res. Ther..

[34] Guzman-Martinez G., Maranon C. (2022). Cyted Ribles Network. Immune Mechanisms Associated with Cardiovascular Disease in Systemic Lupus Erythematosus: A Path to Potential Biomarkers. Front. Immunol..

[35] Melamud M.M., Ermakov E.A., Boiko A.S., Parshukova D.A., Sizikov A.E., Ivanova S.A., Nevinsky G.A., Buneva V.N. (2022). Serum Cytokine Levels of Systemic Lupus Erythematosus Patients in the Presence of Concomitant Cardiovascular Diseases. Endocr. Metab. Immune Disord. Drug Targets.

[36] Li J., Fu Q., Cui H., Qu B., Pan W., Shen N., Bao C. (2011). Interferon-A Priming Promotes Lipid Uptake and Macrophage-Derived Foam Cell Formation: A Novel Link between Interferon-A and Atherosclerosis in Lupus. Arthritis Rheum..

[37] Gegenava T., Gegenava M., Steup-Beekman G.M., Huizinga T.W.J., Bax J.J., Delgado V., Marsan N.A. (2020). Left Ventricular Systolic Function in Patients with Systemic Lupus Erythematosus and Its Association with Cardiovascular Events. J. Am. Soc. Echocardiogr..

[38] Divard G., Abbas R., Chenevier-Gobeaux C., Chanson N., Escoubet B., Chauveheid M.P., Dossier A., Papo T., Dehoux M., Sacre K. (2017). High-Sensitivity Cardiac Troponin T Is a Biomarker for Atherosclerosis in Systemic Lupus Erythematous Patients: A Cross-Sectional Controlled Study. Arthritis Res. Ther..

[39] Sabio J.M., Los Ríos C.G.-D., Medina-Casado M., Del Mar Del Águila-García M., Cáliz-Cáliz R., Díaz-Chamorro A. (2023). High-Sensitivity Cardiac Troponin I Is a Biomarker for Increased Arterial Stiffness in Systemic Lupus Erythematous Women with Normal Kidney Function. Rheumatol. Int..

[40] Shao K., Yuan F., Chen F., Wang J., Shao X., Zhang F., Zhu B., Wang Y. (2023). Assessing Myocardial Involvement in Systemic Lupus Erythematosus Patients without Cardiovascular Symptoms by Technetium-99m-Sestamibi Myocardial Perfusion Imaging: A Correlation Study on Nt-Probnp. Curr. Med. Imaging.

[41] Parperis K., Velidakis N., Khattab E., Gkougkoudi E., Kadoglou N.P.E. (2023). Systemic Lupus Erythematosus and Pulmonary Hypertension. Int. J. Mol. Sci..

[42] Kadoglou N.P.E., Parissis J., Karavidas A., Kanonidis I., Trivella M. (2022). Assessment of Acute Heart Failure Prognosis: The Promising Role of Prognostic Models and Biomarkers. Heart Fail. Rev..

[43] Sacre K., Vinet E., Pineau C.A., Mendel A., Kalache F., Grenier L.P., Huynh T., Bernatsky S. (2024). N-Terminal Pro-Brain Natriuretic Peptide Is a Biomarker for Cardiovascular Damage in Systemic Lupus Erythematous: A Cross-Sectional Study. Rheumatology.

[44] Nguyen T.H.P., Fagerland M.W., Hollan I., Whist J.E., Feinberg M.W., Agewall S. (2023). High-Sensitivity Cardiac Troponin T Is Associated with Disease Activity in Patients with Inflammatory Arthritis. PLoS ONE.

[45] El-Gamasy M.A., Elsalam M.M.A., Latif A.M.A.-E., Elsaid H.H. (2019). Predictive Values of Dyslipidemia and B-Type Natriuretic Peptide Levels in Juvenile Systemic Lupus Erythematosus: A Two Center-Experience. Saudi J. Kidney Dis. Transpl..

[46] Dong X., Zhao Y., Zhao Z., Fang J., Zhang X. (2023). The Association between Marathon Running and High-Sensitivity Cardiac Troponin: A Systematic Review and Meta-Analysis. J. Back. Musculoskelet. Rehabil..

[47] Tiwari D., Aw T.C. (2023). Optimizing the Clinical Use of High-Sensitivity Troponin Assays: A Review. Diagnostics.

[48] Parissis J.T., Papadakis J., Kadoglou N.P., Varounis C., Psarogiannakopoulos P., Rafouli-Stergiou P., Ikonomidis I., Paraskevaidis I., Dimopoulou I., Zerva A. (2013). Prognostic Value of High Sensitivity Troponin T in Patients with Acutely Decompensated Heart Failure and Non-Detectable Conventional Troponin T Levels. Int. J. Cardiol..

